# Case Report: Sudden Fatal Hemorrhage in Ulcerative Fungal Laryngotracheitis—A Pediatric Case Report

**DOI:** 10.3389/fped.2021.764027

**Published:** 2022-01-11

**Authors:** Andrea Porzionato, Elena Stocco, Aron Emmi, Veronica Macchi, Raffaele De Caro

**Affiliations:** Section of Human Anatomy, Department of Neuroscience, University of Padova, Padova, Italy

**Keywords:** invasive aspergillosis, *Aspergillus* laryngotracheitis, laryngotracheal ulcers, inferior thyroid artery, hemoptysis, acute lymphoblastic leukemia

## Abstract

In this report, we describe an autopsy case of a child affected by acute lymphoblastic leukemia and opportunistic pulmonary aspergillosis. The patient died because of a full-thickness tracheal wall ulceration with right inferior thyroid artery lesion and sudden hemorrhage, likely ascribable to undiagnosed invasive *Aspergillus* laryngotracheitis. *Aspergillus* infection, particularly in immunocompromised patients, should be considered an urgent risk factor to manage as it may lead to sudden fatal events in absence of evident critical symptoms.

## Introduction

*Aspergillus species* (*spp.)* are responsible for opportunistic infections inducing a wide spectrum of diseases (sinusitis, bronchitis, allergic bronchopulmonary aspergillosis, aspergilloma, invasive aspergillosis) whose severity is related to the host's immunity (1, 2). Specifically, among immunocompromised patients, invasive pulmonary aspergillosis is the most common clinical manifestation, leading to severe life-threatening conditions, high morbidity/mortality and healthcare costs (3–5). The infection is typically associated with pulmonary parenchymal invasion, inflammation, and possible hematogenous spread (1). The risk for its occurrence increases along with the extent/duration of neutropenia, coexistence of other pathological conditions (i.e., solid/hematological malignancies, human immunodeficiency virus (HIV) infection, graft-versus-host disease), or specific therapies/clinical approaches (i.e., corticosteroid or immunosuppressive agents, chemotherapy, hematopoietic stem cell or solid organ transplantation) (6–9). Prompt initiation of systemic/local antifungal therapy and restoration of host defenses are fundamental to improve outcomes and patient survival. In fact, once the respiratory failure occurs, the prognosis is poor (10–13).

An uncommon variant of invasive pulmonary aspergillosis is *Aspergillus* laryngotracheobronchitis (1, 14–16), which, in turn, can be subdivided into three types: obstructive, showing massive intraluminal growth of *Aspergillus ssp*. with the presence of thick mucus plugs; pseudomembranous necrotizing, showing formation of whitish pseudomembranes characterized by the presence of hyphae, fibrin, and necrotic debris; and ulcerative, characterized by focal lesions penetrating the tracheobronchial wall with possible formation of bronchoesophageal or bronchoarterial fistulas (14, 17, 18). These three variants may coexist in different portions of the laryngo-tracheobronchial tract or may represent different stages of disease development (18). Moreover, their diagnosis may be difficult due to non-specific clinical manifestation (especially in the early stages) and lacking in typical radiographic findings (9). Direct bronchoscopy for airway visualization combined with bronchial biopsy is reported to be the gold standard for early identification of the infection. Unfortunately, the procedure is not free from risks because of possible hemorrhage associated with the removal of the infected material (6, 12, 19). Death is usually due to multiple organ failure or airway obstruction and acute respiratory distress syndrome (ARDS) (6).

In this scenario, larynx aspergillosis is an extremely rare condition being usually an expression of a widespread infection rather than an isolated process (20). It occurs as dissemination from lower airways (21), secondary to pulmonary involvement (22), invasive aspergillosis of the tracheobronchial tree (23) and bronchopulmonary disease (24, 25).

To the best of our knowledge, we here report the first autopsy case of a pediatric immunocompromised patient likely affected by invasive *Aspergillus* laryngotracheitis, who suddenly died of hemorrhage caused by full-thickness ulceration of the tracheal wall with injury of the right inferior thyroid artery.

## Case Description

A child of age below 6 years old was diagnosed for pre-B acute lymphoblastic leukemia, rearranged TEL/AML = t (12; 21), SNC1; hence, chemotherapy started according to the international collaborative treatment protocol AIEOP2017. At day 52 after the start of chemotherapy, fever occurred (38.6°C) (also accompanied by a 20-day catarrhal cough), and it was managed with Piperacillin-Tazobactam therapy. Diagnostic insights also revealed positivity only for Rhinovirus in the superficial respiratory secretions. At day 63, because of good general conditions, the patient was discharged; however, at day 66, the child was accompanied to the emergency department for fever recurrence. Bone marrow aplasia with very severe leukopenia (0.3 × 10^3^/μL), thrombocytopenia (7 × 10^3^/μL), and anemia (hemoglobin: 7.0 g/dL) were diagnosed, requiring hospitalization in pediatric onco-hematology. Positivity for *Aspergillus* and pan-fungal antigens (i.e., galatomannan and beta-D-glucan, respectively) was detected (Table 1); mycete culture in nasopharyngeal aspirate and peripheral blood were negative. Cytomegalovirus (CMV) presence was also shown. Additionally, in a clinical setting characterized by the persistence of medullary aplasia, a computed tomography (CT) scan showed the presence of a worsening interstitial pneumonia. Therapy consisted of antibiotics (Piperacillin-Tazobactam), antivirals (Foscavir), and antifungals (Amphotericin B); transfusions of both platelets and concentrated red blood cells were also performed, together with granulocyte colony-stimulating factor administration. Specifically, as regards antifungal therapy, Amphotericin B dosage was adjusted over time (Table 1): from 3 mg/kg/day (days 69–76) to 5 mg/kg/day (days 77–82). At day 83, the child was transferred to the pediatric intensive care unit; positivity for aspergillary and pan-fungal antigens together with the presence of CMV-DNA persisted. The dosage of Amphotericin B therapy was increased to 6.25 mg/kg/day. At day 90, responsiveness to antibiotic/antiviral/antifungal treatments led to a significant improvement of the clinical picture; however, 3 days later, following a sudden cough, the child presented a very abundant bleeding from the oral cavity initially supposed to be of gastric origin. The hemorrhage required resuscitation with ventilation, tracheal intubation, and external cardiac massage for the onset of extreme bradycardia followed by asystole. Despite the resuscitation maneuvers, the infusion of fluids, adrenaline, and bicarbonate, the child died (Table 1).

**Table 1:** Timeline showing the disease course of the patient up to death.

**Days from diagnosis**	**Clinical history**
1	Starts chemotherapy according to AIEOP2017 protocol
52–55	Day 52, fever and hospitalization Catarrhal cough; positivity only for Rhinovirus in respiratory secretions
59–62	Persistent positivity for Rhinovirus in respiratory secretions
63	Good general conditions and discharge
66	Pediatric emergency department entrance for fever Diagnosis of medullary aplasia Transfer to the department of pediatric onco-hematology
69–76	Amphotericin B, 3 mg/kg/day
77	*Aspergillary antigen (galactomannan) – Positive, serum*
77–82	Amphotericin B, 5 mg/kg/day
80	*Aspergillary antigen (galactomannan) – Positive, serum* *Panfungal antigen (Beta-D-glucan) (> 500 pg/ml) – Positive, serum*
83	Pediatric intensive care transfer *Aspergillary antigen (galactomannan) – Positive, serum* *Panfungal antigen (Beta-D-glucan) (369 pg/ml) – Positive, serum*
83-onward	Amphotericin B, 6.25 mg/kg/day
88	*Panfungal antigen (Beta-D-glucan) (130 pg/ml) – Positive, serum*
94	Sudden hemorrhage and fatal outcome
AUTOPSY	Diagnosis of ulcerative *Aspergillus* laryngotracheitis

**Figure 1:**
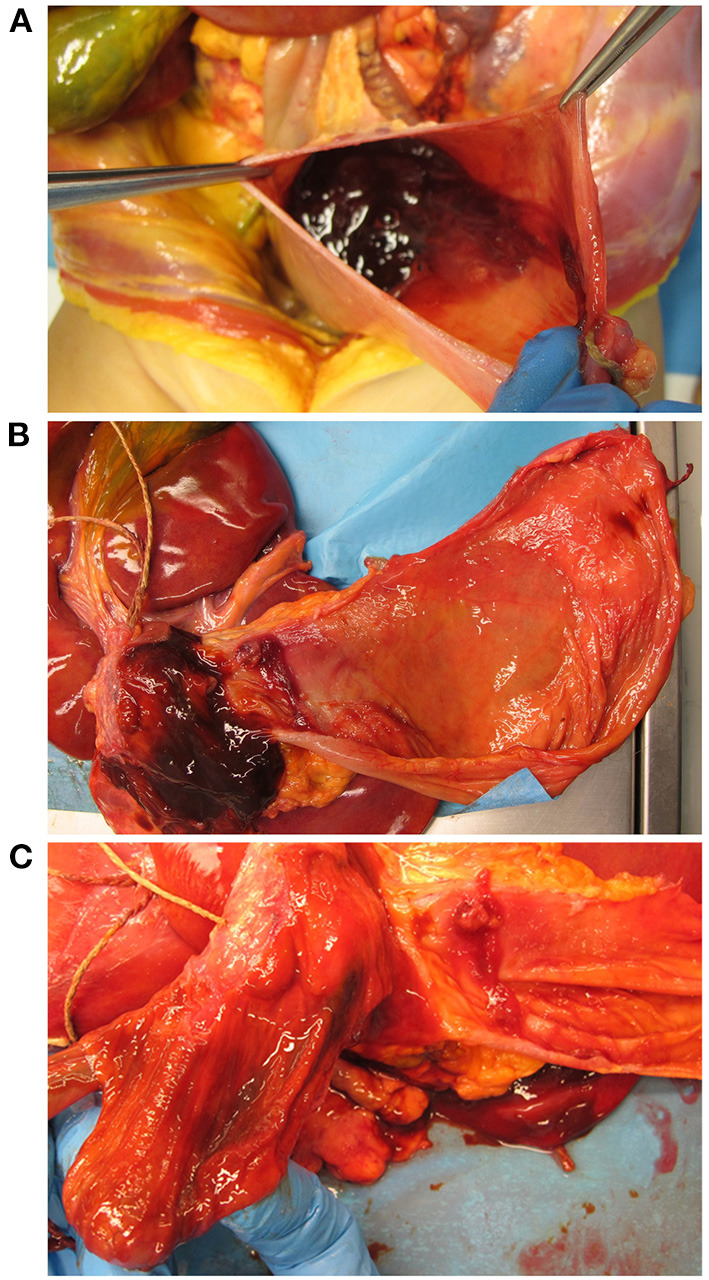
Macroscopic examination of the gastric, pyloric, and duodenal mucosa. Opening of the stomach showing presence of a brownish gelatinous material inside, **(A)** which, once removed, revealed an intact mucosa without signs of lesions **(B)**. Presence of brownish gelatinous material in the duodenum **(B)** with no sign of ulcerative alteration of the pyloric and duodenal mucosa **(C)**.

At autopsy, blood presence was observed in the lumen of the upper tracts of the alimentary canal (i.e., the oral cavity, pharynx, esophagus, stomach, and duodenum) in the absence of wall injury (Figures 1A–C).

**Figure 2:**
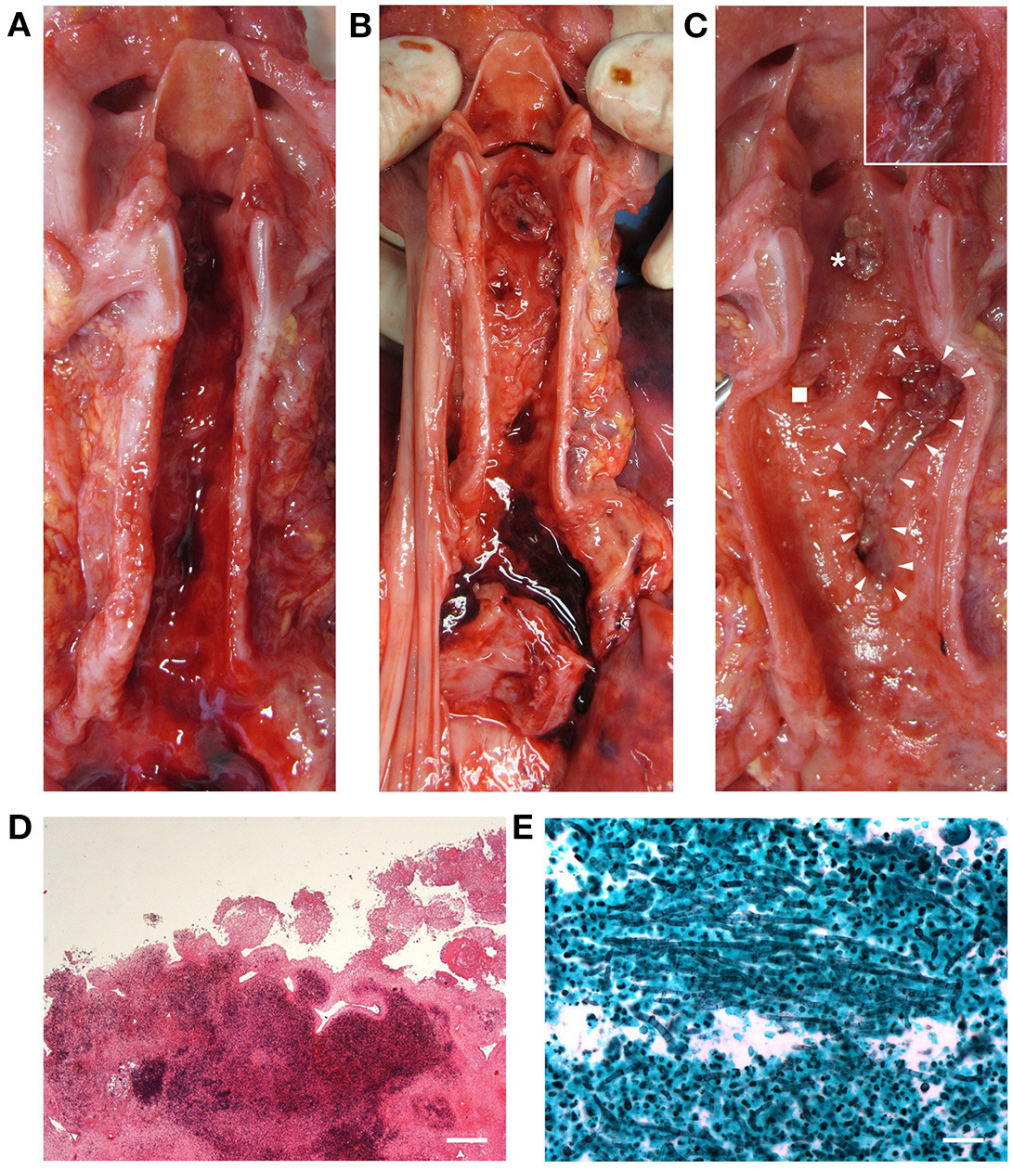
Macroscopic examination of the laryngeal cavity and tracheal lumen. Opening of the larynx, trachea, and bronchi with evidence of blood **(A)**. Laryngotracheal ulcerative lesions with presence of some necrotic pseudomembranous-like features (asterisk, cricoid lesion; square, left anterolateral tracheal lesion; triangles, right anterolateral tracheal lesion) consistent with invasive *Aspergillus* laryngotracheitis **(B,C)**; the insert in **(C)** shows a magnification of the site corresponding to tracheal wall perforation and thyroid artery damage. Histopathologic examination by hematoxylin and eosin of the laryngeal mucosa at the site of ulceration (transversal section), showing inflammatory infiltration and signs of tissue necrosis (Scale bar: 400 μm) **(D)**. Grocott–Gomori's methenamine silver stain showing *Aspergillus* branching hyphae (black) infiltrating the laryngotracheal wall in a necrotic ground **(E)** (Scale bar: 50 μm).

Considering the laryngotracheal tract, three ulcerative lesions with destruction of the cartilage components were observed; necrotic pseudomembranous features were partly also recognized (Figures 2A–D). Specifically, the ulcers were located at the anterior aspect of the cricoid cartilage (circular appearance, 1 cm in diameter); at the left antero-lateral aspect of the first tracheal ring (circular appearance, 1 cm in diameter); at the right antero-lateral side of the trachea along the first tracheal rings (oriented according to the tracheal axis, 1 × 4.5 cm) (Figures 2A–C). Death occurred as a consequence of right inferior thyroid artery lesion due to full-thickness ulcerative perforation of the tracheal wall. An artery protruded at the cranial end of the right anterolateral tracheal ulcer (Figure 2C, insert).

**Figure 3:**
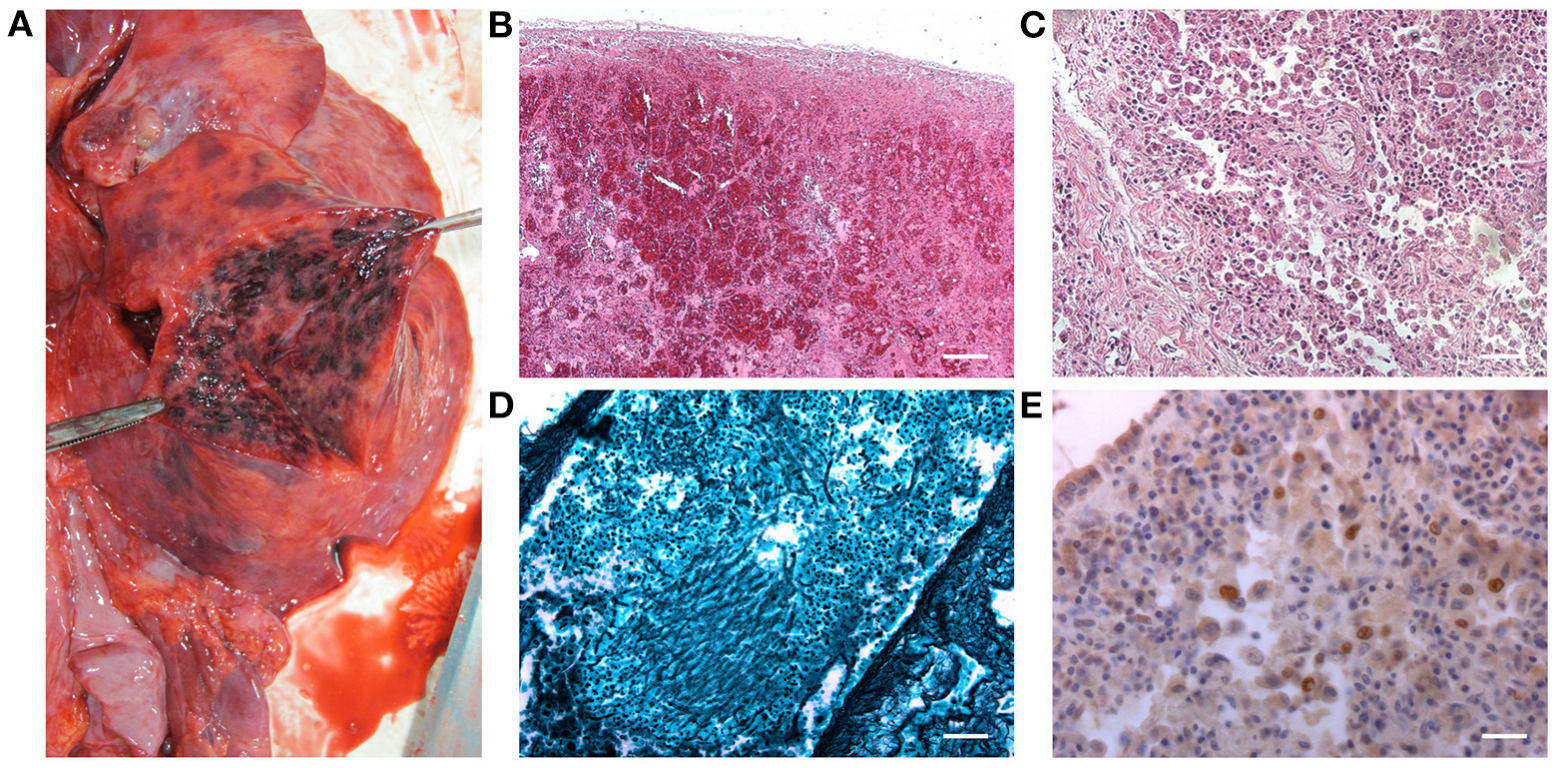
Macroscopic appearance of the inferior lobe of the right lung after surface incision **(A)**. Histopathological characterization by hematoxylin and eosin stain of the right lung parenchyma: hemorrhagic invasion of the air spaces **(B)** and the typical appearance of the tissue in interstitial pneumonia **(C)** were detected [Scale bars: 200 μm **(B)**; 50 μm **(C)**]. Grocott–Gomori's methenamine silver stain showing *Aspergillus* branching hyphae (black) infiltrating the lung parenchyma **(D)** (Scale bar: 50 μm). Immunohistochemical staining for CMV, highlighting the presence of immunoreactive cells **(E)** (Scale bar: 25 μm).

Macroscopic examination of the lungs showed patchy black/red areas of hemorrhagic invasion of airspaces (Figure 3A).

The laryngo-tracheal lesions and the lungs were sampled, fixed in 10% buffered formalin, and processed for histopathological analyses. Specifically, all tissues underwent hematoxylin and eosin staining (Figures 2D, 3B,C, respectively) and the presence of hyphae was detected by Grocott–Gomori's methenamine silver (GMS) staining (Figures 2E, 3D, respectively). In parallel, immunoperoxidase staining was performed on a Dako EnVision Autostainer according to manufacturer recommendations; antibody for E-13 CMV (monoclonal mouse anti-human, aurogene, code number: 11-003) was used to detect presence of eventual positive cellular elements. Regarding the lung tissue, focal areas of necrosis, likely associated to *Aspergillus* infection (as confirmed by hyphae presence) and interstitial pneumonia evidence, consistent with CMV etiopathogenesis, were observed; hemorrhagic invasion of airspaces was also found (Figures 3B–E).

## Discussion

Invasive *Aspergillus* laryngotracheitis is a rare clinical variant of invasive pulmonary aspergillosis, characterized by local invasion of the laryngotracheal wall by *Aspergillus spp*. As it typically affects immunocompromised patients, prolonged neutropenia is recognized among the main risk factors for its onset (18, 26–28). Considering the typical clinical manifestations associated with invasive laryngotracheal aspergillosis (obstructive/pseudomembranous/ulcerative elements), their coexistence may occur in the laryngo-tracheobronchial tract, thus leading to a complex clinical picture (18).

Revising the literature, ulcerative disruption of respiratory tract, descending from invasive *Aspergillus* laryngotracheal infection, are reported in adults [e.g., (9, 10, 14, 20, 25, 29–46)] and in adolescents (15 and 17 years old) (47, 48) but not in pediatric patients. According to our knowledge, only Barnes et al. (49) and Athanassiadou et al. (50) described *Aspergillus* laryngotracheobronchitis in children (6 and 2 years old) affected by acute lymphocytic leukemia; however, in these cases, the characterizing elements included plaques and necrotic cells, whereas ulcerative lesions were not reported. Moreover, the infection positively resolved with proper antifungal treatment without recurrence.

Here, we describe for the first time to our knowledge, a fatal hemorrhagic event caused by a previously undiagnosed ulcerative *Aspergillus* laryngotracheitis in an immunocompromised child. On examination, the presence of ulcerative lesions, also displaying necrotic tissue evidence, were recognized in the larynx and trachea. Specifically, the histopathological analysis showed compromised cartilage integrity and, more surprisingly, a full thickness perforation of the tracheal wall. This event led to right inferior thyroid artery damage followed by sudden hemorrhage and death.

Revising the literature, complete tracheal wall perforation by *Aspergillus* was described by Gonzalez et al. (48) reporting about an immunocompromised adolescent (recurrent precursor B-cell acute lymphoblastic leukemia) showing a 15 ×15 mm large defect of the right-sided distal tracheal wall on the membranous pars; communication with the right pleural cavity was established. In that case, the perforation was successfully repaired and resolved using a pedicle muscular flap (*latissimus dorsi*) and temporary airway stenting. Additionally, Swiss et al. (44) reported about an invasive *Aspergillus* laryngopharyngitis affecting an immunocompromised woman (history of myelodysplastic syndrome and acute myelogenous leukemia) and leading to autolaryngectomy: laryngeal destruction up to complete laryngotracheal separation were observed. The patient survived by aggressive antifungal therapy and surgical debridement. Only fatal hemorrhage amenable to necrotizing tracheobronchial aspergillosis was referred by Berlinger and Freeman (10). A 21-year-old immunocompromised man (acute lymphoblastic leukemia) died of a profuse and uncontrollable bleeding due to a fistula in the bronchus intermedius, which, in turn, compromised the right pulmonary artery. To our knowledge, the other case reports describing ulcers in *Aspergillus* laryngo-tracheobronchitis were mainly all positively managed with disease resolution. As for the fatal cases, death occurred for exacerbation of the concomitant disease (20, 36, 39) or complications secondary to its therapy (i.e., bone marrow transplantation) (47), respiratory failure, ARDS, and pulmonary disease (9, 10, 38, 41–43) up to cardiopulmonary arrest (29).

The specific elements (pediatric age) and the clinical findings (larynx involvement; full-thickness tracheal perforation; thyroid artery damage; death for sudden hemorrhage) here reported confirm the unicity of this autopsy case showing features descending from fungal infection/invasion (as confirmed by GMS staining) and that may be likely ascribed to ulcerative *Aspergillus* laryngotracheitis. The most frequent complaints associated to *Aspergillus* laryngotracheobronchitis include productive cough, fever, dyspnea, chest pain, and hemoptysis (9, 33, 51); it is likely that both the cough and recurrent vomiting played a fundamental role in worsening the severity of the laryngotracheal lesions, thus inducing the fatal bleeding event. Additionally, the severe thrombocytopenia, characterizing the bone marrow aplasia, furtherly aggravated the hemorrhagic syndrome as consequence of hemostasis alteration. In this context, the severity of the clinical conditions described may also be further worsened by CMV interstitial pneumonia whose presence is prevalent in immunocompromised hosts; according to the literature, immunocompromised children are expected to develop a pneumonic process in about 80% of the cases (52). Specifically, clinical presentation of CMV interstitial pneumonia includes cough, increased work of breathing, hypoxemia, diffuse adventitious lung sounds and persistent fever too (53, 54).

Although *Aspergillus* was probably involved in the present case, other thin septate fungi could also be considered in differential diagnosis, such as *Penicillium* and *Talaromyces*. For instance, *Talaromyces marneffei* (formerly *Penicillium marneffei*), despite mainly affecting HIV-positive patients, triggers symptoms similar to that here described (fever, cough, and dyspnea) in HIV-negative patients. However, only a few cases were reported in presence of hematological malignancies in children (55). Additionally, this infection is typically associated to Southeast Asia regions (and not Europe) (56, 57).

In conclusion, invasive *Aspergillus* may trigger aggressive pathological conditions, especially in immunocompromised patients; multiple sites of infection may coexist, and they can be also characterized by different specific features. Apart from these physio-pathological and clinical considerations, it is also important to stress that in this case the ulcerative tracheobronchitis was only diagnosed at autopsy and the hemorrhagic event occurred in a quite surprising way for the physicians. Unfortunately, diagnosis of *Aspergillus* laryngotracheitis is particularly difficult due to its rarity and ambiguous symptoms presence, especially in pediatric patients. It descends that pediatric clinical practice on invasive *Aspergillus ssp*. related diseases largely derives from data gathered in adult subjects, itself suffering from significant gaps on prevention, diagnosis, and treatment (5).

Considering the clinical setting described, further elements have been provided for more consciousness on eventual fatal consequences ascribable to invasive aspergillosis. A high index of suspicion should be adopted in case of *Aspergillus* opportunistic infection in particularly weak patients. In fact, early infection identification and rapid therapy initiation undoubtedly represent the keystones to avoid unexpected ominous outcomes with safe resolution (58). Additionally, multiple diagnostic approaches should be considered due to possible different sensitivities of culture and antigen tests (59). In the present case, for instance, mycete cultures were negative, although in the presence of several antigen tests positivities (i.e., galatomannan and beta-D-glucan, respectively). In accordance with Kuo et al. (20), bronchoscopy should be recommended in immunocompromised patients showing signs of airway hoarseness, unexplained sore throat, or obstruction to guarantee adequate clinical management and avoid poor prognosis: reestablishment of immune function is mandatory for good outcomes. Physicians should be better aware about the risk of ulcerative *Aspergillus* laryngotracheitis in immunocompromised patients and the possibility of rapidly fatal hemorrhagic evolution although rare.

## Data Availability Statement

The original contributions presented in the study are included in the article/supplementary material, further inquiries can be directed to the corresponding author/s.

## Ethics Statement

Ethical review and approval was not required for the study on human participants in accordance with the local legislation and institutional requirements. Written informed consent from the participants' legal guardian/next of kin was not required to participate in this study in accordance with the national legislation and the institutional requirements.

## Author Contributions

AP and RDC performed the autopsy, identified the case, and sampled the tissues. AE performed the histological and immunohistochemical analyses. RDC, AP, and VM were responsible of data interpretation, clinical correlations, and performed the final supervision of the manuscript. AP and ES conducted the literature search. ES prepared the first draft of the manuscript. All authors contributed to the article and approved the submitted version.

## Conflict of Interest

The authors declare that the research was conducted in the absence of any commercial or financial relationships that could be construed as a potential conflict of interest.

## Publisher's Note

All claims expressed in this article are solely those of the authors and do not necessarily represent those of their affiliated organizations, or those of the publisher, the editors and the reviewers. Any product that may be evaluated in this article, or claim that may be made by its manufacturer, is not guaranteed or endorsed by the publisher.
